# Molecular characterization of *G6PD* mutations reveals the high frequency of G6PD Aures in the Lao Theung population

**DOI:** 10.1186/s12936-020-03560-7

**Published:** 2021-01-07

**Authors:** Amkha Sanephonasa, Chalisa Louicharoen Cheepsunthorn, Naly Khaminsou, Onekham Savongsy, Issarang Nuchprayoon, Kamonlak Leecharoenkiat

**Affiliations:** 1 Oxidation in Red Cell Disorders Research Unit, Department of Clinical Microscopy, Faculty of Allied Health Sciences, Chulalongkorn University, 154, Rama I Road, Patumwan District, 10330 Bangkok, Thailand; 2 Department of Biochemistry, Faculty of Medicine, Chulalongkorn University, Bangkok, Thailand; 3 Department of Medical Laboratory, Faculty of Medical Technology, University of Health Sciences, Vientiane, Lao PDR; 4 Department of Pediatrics, Faculty of Medicine, Chulalongkorn University, Bangkok, Thailand

**Keywords:** G6PD genotype, G6PD deficiency, *G6PD* mutation, G6PD Aures, Laos, Lao Theung

## Abstract

**Background:**

The prevalence and genotypes of G6PD deficiency vary worldwide, with higher prevalence in malaria endemic areas. The first-time assessment of G6PD deficiency prevalence and molecular characterization of *G6PD* mutations in the Lao Theung population were performed in this study.

**Methods:**

A total of 252 unrelated Lao Theung participants residing in the Lao People’s Democratic Republic (PDR) were recruited. All participant samples were tested for G6PD enzyme activity and *G6PD* gene mutations. The amplification refractory mutation system (ARMS)-PCR for detecting G6PD Aures was developed.

**Results:**

The *G6PD* mutations were detected in 11.51% (29/252) of the participants. Eight G6PD mutations were detected. The G6PD Aures was the most common mutation identified in this cohort, which represented 58.62% (17/29) of all mutation. The mutation pattern was homogenous, predominantly involving the G6PD Aures mutation (6.75%), followed by 1.19% G6PD Union and 0.79% each G6PD Jammu, G6PD Mahidol and G6PD Kaiping. One subject (0.4%) each carried G6PD Viangchan and G6PD Canton. Interestingly, one case of coinheritance of G6PD Aures and Quing Yan was detected in this cohort. Based on levels of G6PD enzyme activity, the prevalence of G6PD deficiency in the Lao Theung population was 9.13% (23/252). The prevalence of G6PD deficient males and females (activity < 30%) in the Lao Theung population was 6.41% (5/78) and 1.72% (3/174), respectively, and the prevalence of G6PD intermediate (activity 30–70%) was 5.95% (15/252).

**Conclusions:**

The G6PD Aures mutation is highly prevalent in the Lao Theung ethnic group. The common G6PD variants in continental Southeast Asian populations, G6PD Viangchan, Canton, Kaiping, Union and Mahidol, were not prevalent in this ethnic group. The technical simplicity of the developed ARMS-PCR will facilitate the final diagnosis of the G6PD Aures.

## Background

Glucose 6˗phosphate dehydrogenase (G6PD) deficiency is the most common hereditary enzymopathy affecting people worldwide. G6PD is a key enzyme in the pentose phosphate pathway (PPP), a metabolic pathway that produces nicotinamide adenine dinucleotide phosphate (NADPH) to maintain the level of a reduced glutathione, which is important for defending against oxidative damage in red blood cells [1, 2]. The gene encoding the G6PD enzyme is located near the telomeric region of the distal arm of the *X* chromosome and is composed of 13 exons and 12 introns [3]. G6PD deficiency is mostly caused by a single nucleotide mutation that gives rise to an amino acid substitution in an exon of the *G6PD* gene, resulting in reduced enzyme activity and stability [4, 5]. G6PD deficiency is an X-linked genetic condition; therefore, this condition is predicted to be more common in males than in females and is expressed in hemizygous males and homozygous or heterozygous females [6].

Most people with G6PD deficiency do not exhibit symptoms unless exposed to an oxidation-inducing agent or an infection [7]. G6PD deficiency causes a spectrum of acute or chronic hemolysis in affected individuals and causes neonatal jaundice, which can result in life-threatening kernicterus in newborns [8]. Persons with G6PD deficiency are more susceptible to red blood cell destruction when exposed to oxidative stress; these agents include oxidant drugs (e.g., sulfones and 8-aminoquinoline) and food (fava beans) [9]. Acute haemolysis due to anti-malarial drugs is a health concern in Southeast Asia [8]. However, 8-aminoquinoline anti-malarials, such as primaquine, remain the only effective drug against relapse caused by *Plasmodium vivax* and *Plasmodium ovale* [10]. Beyond anti-malarial drugs, G6PD deficiency is also relevant to other medicines and infections, such as dengue or hepatitis virus, and has a risk of serious complications resulting from acute renal failure [11, 12].

More than 215 *G6PD* mutations have been reported [3]. The prevalence of G6PD deficiency correlates with the geographical distribution of malaria since this disorder is believed to provide protection against the disease [13, 14]. Currently, more than 400 million people worldwide carry an abnormal *G6PD* gene, particularly in Asia, Africa, the Middle East and the Mediterranean [7, 15, 16]. *G6PD* genotypes show population specificity. For example, in Southeast Asia, the G6PD Mahidol mutation is predominant among the Myanmar and Mon populations [17], while the G6PD Viangchan mutation is common among the Thai [18], Cambodian [19] and Lao populations [20–25]. No studies have been conducted to ascertain the prevalence of G6PD deficiency in the Mon-Khmer or Lao Theung group. The Lao Theung group is the second largest ethnic group in Laos and has its own culture and language (the Mon-Khmer language) [26]. The Lao Theung group originated from the Austro-Asiatic family and migrated to Southeast Asia in prehistoric times [27]. In addition to the Lao PDR, Lao Theung individuals live in Thailand, China, Myanma, Cambodia and Vietnam [28]. This study aimed to assess the prevalence and perform molecular characterization of G6PD deficiency in the Lao Theung ethnic group residing in the Lao PDR. Since severe acute haemolytic anaemia can be triggered by G6PD deficiency, addressing the prevalence and molecular characterization of *G6PD* mutations in the Lao Theung ethnic group is an important public health issue.

## Methods

### Participants

Peripheral blood samples were collected from 252 unrelated healthy Lao Mon-Khmer people, including 174 females and 78 males. Participants’ ages ranged from 18 to 50 years old. The participants were living in the Feuang District, Vientiane Province, and Lao PDR. This study was a community-based survey which was approved by the Ethics Committee of the university of Health Sciences, Lao PDR. The sample collection was performed in December 2016. Written informed consent was obtained before blood sampling from all individual participants included in the study.

### G6PD enzyme activity assay

The blood samples were stored at 4 ºC and analysed for G6PD enzyme activity within 7 days after collection. All samples were measured for G6PD enzyme activity in duplicate using a quantitative G6PD kit (Trinity G-6-PDH Kit, Trinity Biotech, Bray, Ireland). The assay was carried out at 30 ºC, and the method was performed following the manufacturer’s instructions. The haemoglobin (Hb) concentration was determined using a HemoCue Haemoglobin System (HemoCue Hb 201 Analyzer, Fisher Scientific, Inc, Waltham, MA, USA). The G6PD enzyme activity values were calculated in units per gram of Hb (U/g Hb).

### Identification of 8 common G6PD Asian types

All 252 subjects were genotyped for the 8 common *G6PD* mutations detected in Asia using a multiplex allele-specific PCR-based assay (DiaplexC™ G6PD Genotyping Kit (Asian type), SolGent, Daejeon, Korea). The genomic DNA of all blood samples was extracted by using a QIAamp® DNA Blood Kit (Qiagen, Düsseldorf, Germany) according to the manufacturer’s recommended protocol and kept at -80 °C until use. The commercial kit enabled the detection of 8 common *G6PD* mutations, including Vanua Lava c.383 T > C, Mahidol c.487 G > A, Mediterranean c.563 C > T, Coimbra c.592 C > T, Viangchan c.871 G > A, Union c.1360 C > T, Canton c.1376 G > T and Kaiping c.1388 G > A. The PCR mixture contained 12 µl of 2X Multiplex PCR Smart Mix, 2 µl of primer mixture, 20–50 ng DNA template and nuclease- free water to obtain a total volume of 25 µl. The PCR cycling conditions used were as follows: initial denaturation at 95 °C for 15 min, followed by 30 cycles of denaturation at 95 °C for 30 sec, annealing at 60 °C for 30 sec and extension at 72 °C for 40 sec; this was followed by a final extension at 72 °C for 5 min. After PCR amplification, 5 µl of the PCR product and 5 µl of a standard marker were separated by 3% agarose gel electrophoresis, stained with SYBR® Green (Thermo Fisher Scientific, USA) and visualized under UV light. The positive results were validated by PCR sequencing.

### **Identification of*G6PD*gene mutations by PCR sequencing**

PCR sequencing was performed to identify the *G6PD* gene mutation in the G6PD-deficient samples that could not be identified by DiaplexC™ G6PD Genotyping Kit. The entire coding sequence (exons 2–13) of the *G6PD* gene was amplified with specific primers following a protocol previously described [17]. The PCR products were purified using an AccuPre® PCR Purification Kit (*Bioneer*, Daejeon, Korea) and subsequently sequenced by using the Sanger sequencing method (*Bioneer Sequencing* Service, Daejeon, Korea). The sequence of G6PD was analysed by BLAST and compared to the *G6PD* gene mutation with database accession no. NC_000023.11.

### Detection of G6PD Aures by the amplification refractory mutation system (ARMS)-PCR method

**Fig. 1:**
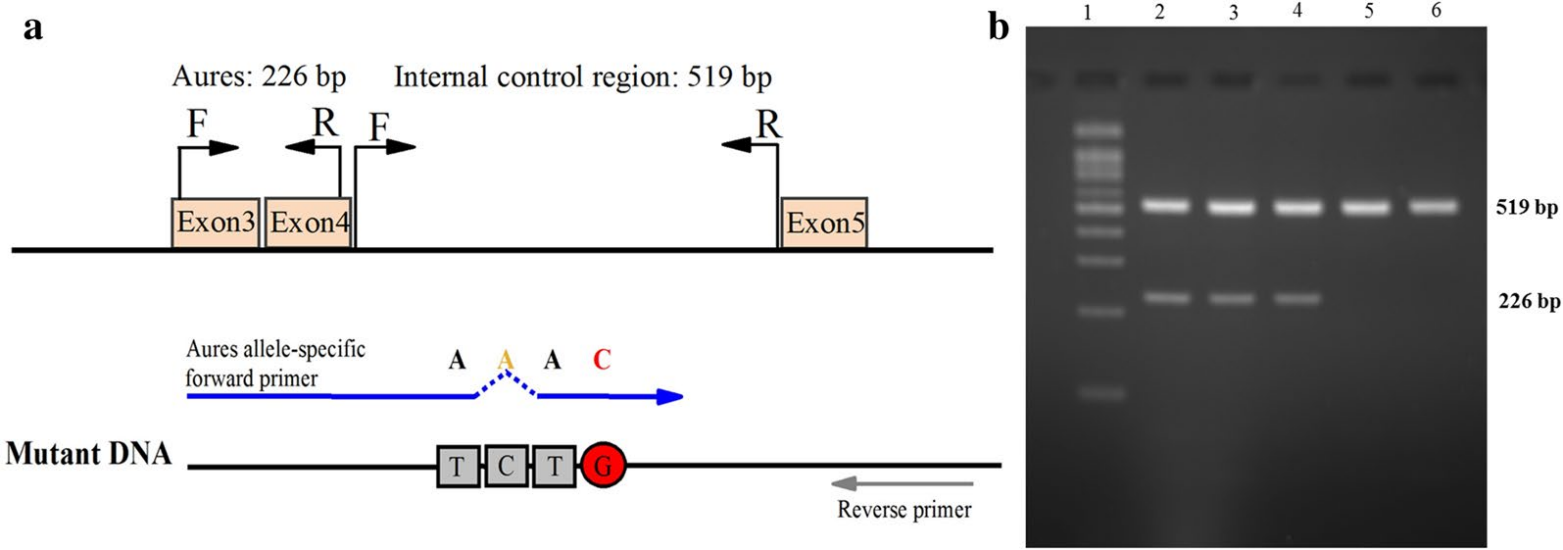
Identification of G6PD Aures by an ARMS-PCR assay. **a** Schematic representation of the primer location and predicted size of PCR products for the G6PD Aures and internal control fragment. **b** Agarose gel electrophoresis represents band of G6PD Aures (226 bp) and internal control fragments (519 bp). Lane 1 represents the 100 bp DNA marker. Lanes 2–4 represent G6PD Aures-positive samples, and lanes 5–6, G6PD Aures-negative samples

The ARMS-PCR method for detecting the G6PD Aures mutation was first developed in this study. The G6PD Aures allele-specific PCR primer pair consisted of a forward primer (5′-ACCTGGCCAAGAAGAAGAT-3′) and a reverse primer (5′-CTCACTCTGTTTGCGGATG-3′), which produce a 226 bp fragment. Two additional primers consisting of a forward primer (5′-TGGTTCTGCCCTCTCTAC-3′) and a reverse primer (5′-GAGACACGGACAGA CAGA-3′) were added to produce a 519 bp fragment of the 3′-UTR region to serve as an internal amplification control (Fig. 1). The multiplex ARMS-PCR mixture contained 2x HotStar Taq Master Mix (Qiagen Multiplex PCR Kit, Qiagen, Düsseldorf, Germany), 200 µM of each dNTP, 10 pmol of each primer, 5× Q-solution and 150 ng genomic DNA. PCR cycling was performed with an initial denaturation at 95 °C for 15 min, followed by 35 cycles of amplification at 95 °C for 30 sec, 55 °C for 1 min and 72 °C for 1 min. The final extension was performed at 72 °C for 5 min. The PCR product was separated on a 1.5% agarose gel and visualized under UV light. The multiplex ARMS-PCR method was validated by direct DNA sequencing. After validation, multiplex ARMS-PCR was used to detect G6PD Aures mutations in all 252 Lao Theung DNA samples. Heterozygosity of the G6PD Aures mutation was identified by direct DNA sequencing.

**Fig. 2:**
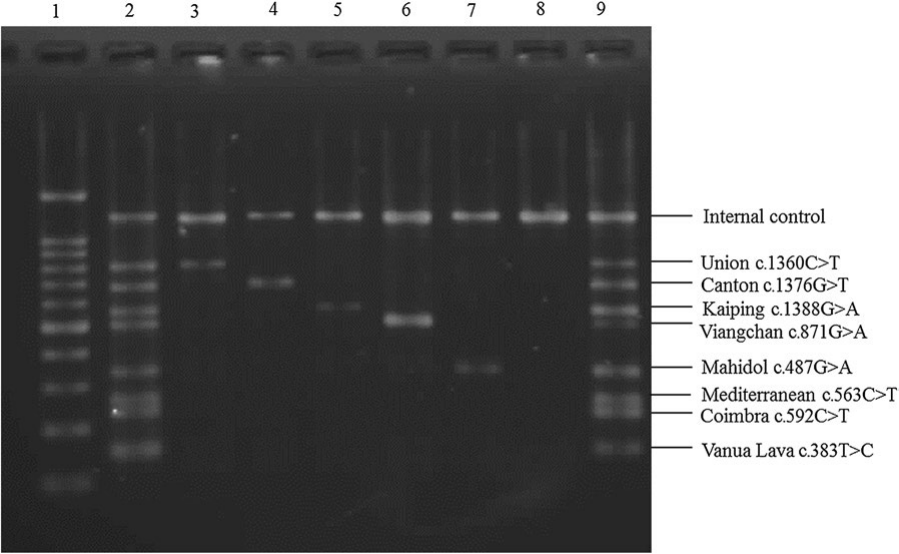
The histogram shows the correlation between G6PD activity (U/gHb) and *G6PD* mutations in the Lao Theung population. **a** G6PD activity (U/gHb) in each *G6PD* mutation group for males. Enzyme activity below 2.0 U/gHb indicates G6PD deficiency; enzyme activity of 2.0 to 4.67 U/gHb indicates G6PD intermediate. All hemizygous subjects presented G6PD deficiency. **b** G6PD activity for the female population. Homozygous subjects presented G6PD deficiency, while the heterozygous subjects have intermediate to normal G6PD enzyme activity

### Data analysis

Data were analysed for the mean, SD, median, and interquartile range (IQR) by using IBM SPSS statistics software version 22 (IBM Corp., Armonk, NY, USA). The 95% confidential intervals were calculated by Java Stat Binomial Confidence Intervals. The adjusted male median G6PD activity was calculated to identify the cut-off values for G6PD deficiency following a calculation method described previously [10]. In detail, the adjusted male median (AMM) values were defined as 100% activity of males after excluding the data of all participants with G6PD activity equal to or less than 10% of the overall median G6PD activity. The cut-off points for G6PD deficiency were median values < 30%of the AMM for G6PD deficient, 30% to 70% of the AMM for G6PD intermediate. Subjects with G6PD activity over 70% of the AMM were defined as having normal activity.

## Results

### Prevalence of ***G6PD*** gene mutations

The overall prevalence of *G6PD* gene mutations in this Lao Theung group was 11.51% (29/252), equating to 6.41% (5/78) in males and 13.79% (24/174) in females. Eight *G6PD* mutation sites were detected (Fig. 2). The most common *G6PD* mutation in this Lao Theung population was G6PD Aures c.143 T > C (6.75%), followed by 1.19% G6PD Union c.1360C > T and 0.79% each G6PD Jammu c.871G > A with nt1311C, G6PD Mahidol c.487G > A and G6PD Kaiping c.1388G > A. One subject (0.4%) each carried G6PD Viangchan c.871G > A and G6PD Canton c.1376G > T (Table 1). Interestingly, one female carried coinherited G6PD Aures c.143 T > C and G6PD Quing Yan c.392G > T mutations was identified in this cohort. The G6PD Vanua Lava c.383 T > C, G6PD Coimbra c.592C > T and G6PD Mediterranean c.563C > T mutations were not detected in this cohort.

**Fig. 3:**
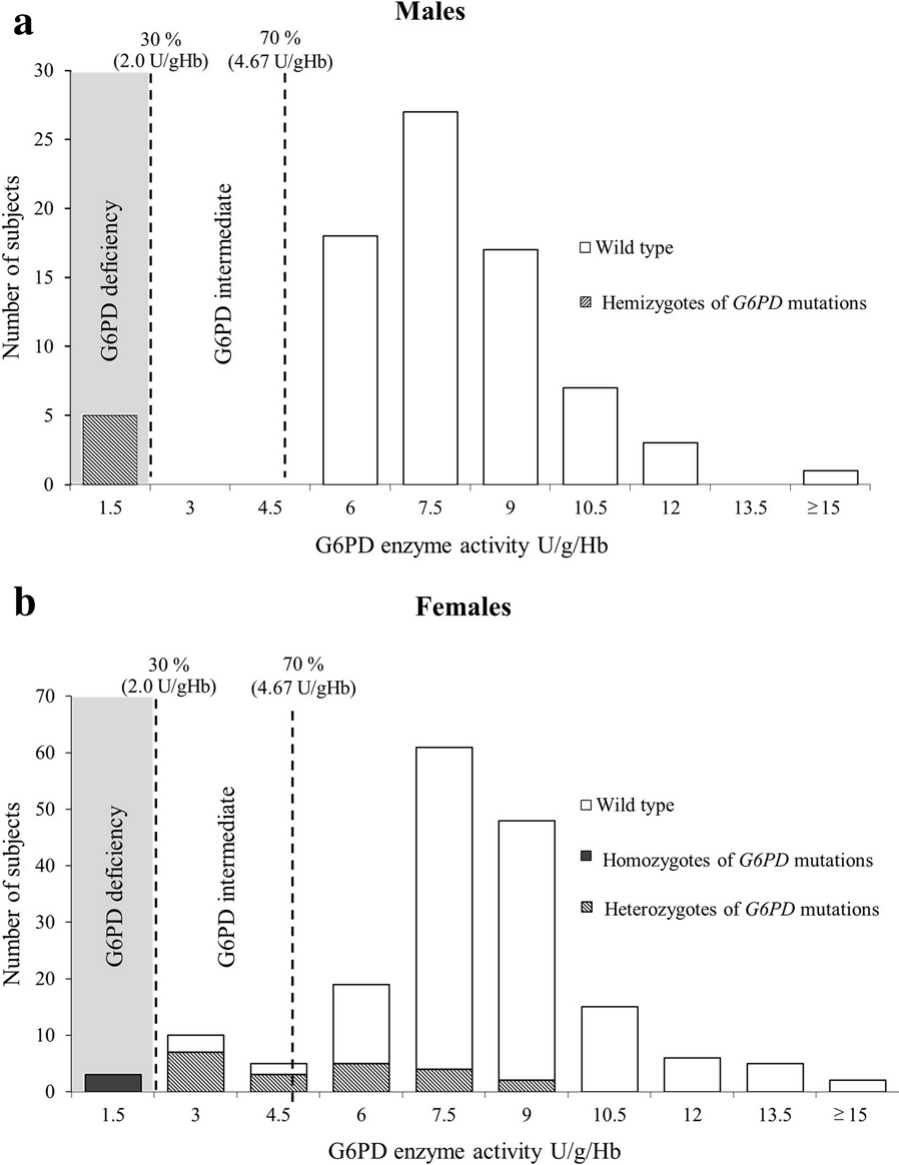
Agarose gel electrophoresis representing 8 G6PD Asian genotypes. Lane 1 represents a 100 bp DNA marker. Lane 2 is a G6PD mutation standard marker. Lanes 3–7 are PCR products of unknown samples, lane 8 is the normal control, and lane 9 is the mutant positive control

**Table 1:** Prevalence and frequency of *G6PD* gene mutations in the Lao Theung ethnic group

Mutation	Normal			Intermediate			G6PD deficient			Prevalence (%)	Frequency (95% Confidence interval)
	M (n = 73)	F (n = 160)		M (n = 0)	F (n = 15)^b^		M (n = 5)	F (n = 4 )			
	Hemi-zygotes	Homo-zygotes	Hetero-zygotes	Hemi-zygotes	Homo-zygotes	Hetero-zygotes	Hemi-Zygotes	Homo-zygotes	Hetero-zygotes		
Viangchan c.871G > A with nt1311T	0	0	0	0	0		1	0	0	1 (0.40)	0.0023 0.0001–0.0130
Jammu c.871G > A with nt1311C	0	0	1	0	0	1	0	0	0	2 (0.79)	0.0047 0.0006–0.0169
Mahidol c.487G > A	0	0	0	0	0	1	0	1	0	2 (0.79)	0.0070 0.0015–0.0204
Union c.1360C > T	0	0	1	0	0	1	1	0	0	3 (1.19)	0.0070 0.0015–0.0204
Kaiping c.1388G > A	0	0	0	0	0	1	1	0	0	2 (0.79)	0.0047 0.0006–0.0169
Canton c.1376G > T	0	0	1	0	0	0	0	0	0	1 (0.40)	0.0023 0.0001–0.0130
Vanua lava c.383 T > C	0	0	0	0	0	0	0	0	0	0	0
Coimbra c.592C > T	0	0	0	0	0	0	0	0	0	0	0
Mediterranean c.563C > T	0	0	0	0	0	0	0	0	0	0	0
Aures c.143 T > C	0	0	8	0	0	6	2	1	0	17 (6.75)	0.0423 0.0252– 0.0660
Quing Yan c.392G > T^a^	ND	ND	ND	0	0	0	0	1	0	1 (0.40)	0.0023 0.0001–0.0130
Total	0	0	11	0	0	10	5	3	0	29 (11.51)	

^a^Coinheritance of G6PD Qing Yan c.392G > T with G6PD Aures c.143 T > C

^b^ Five females with G6PD intermediate can not identify *G6PD* mutation

*ND* not done

### **Prevalence of G6PD deficiency calculated based on the enzyme activity**

G6PD enzyme activity was measured by using a quantitative G6PD kit. The median values of the G6PD enzyme activity for the entire population was 7.13 U/gHb (IQR: 6.08–8.43 U/ gHb), ranging from 0.01 to 25.73 U/ gHb. The median and IQR of the G6PD activity of males and females were 6.68 U/gHb; IQR: 5.85–8.03 U/gHb and 7.21 U/gHb; IQR:6.22–8.34 U/gHb, respectively. The cut-off value for G6PD deficiency (activity below 30% of the AMM) in this cohort was < 2.0 U/gHb (Fig. 3). Enzyme activity between 2.0 and 4.67 U/ gHb (activity 30–70% of the AMM) were considered G6PD intermediate. Enzyme activity greater than 4.67 U/ gHb (activity > 70% of the AMM) were considered G6PD normal activity. Based on the enzyme activity cut-off values, the prevalence of G6PD deficiency (enzyme activity < 70%) in this cohort was found to be 9.13% (23/ 252), comprising 3.17% (8/ 252) of G6PD deficient (enzyme activity < 30%) and 5.95% (15/ 252) of G6PD intermediate (enzyme activity 30–70%). The prevalence of G6PD deficiency (enzyme activity < 30%) among females was 1.72% (3/ 174), while that among males was 6.41% (5/ 78). The median of enzyme activity in the 5 hemizygous deficient males with G6PD deficiency (enzyme activity < 30%) were 0.64 U/gHb; IQR: 0.27–0.89 U/gHb. Three females with G6PD deficiency (range 0.39–1.26 U/ gHb) included 2 homozygous mutation (1 G6PD Mahidol and 1 G6PD Aures) and 1 coinheritance of a heterozygous *G6PD* mutation (G6PD Aures and G6PD Quing Yan). Fifteen female subjects with G6PD intermediate activity consisted 10 heterozygous mutations and 5 subjects cannot identify *G6PD* mutation. Eleven subjects carried heterozygous of the *G6PD* mutation had normal G6PD enzyme activity (Table 1). The hemizygous males (n = 2) and homozygous female (n = 1) for the G6PD Aures mutation presented with a severe G6PD deficiency, whereas the phenotype of G6PD Aures heterozygous females varied from normal to moderately G6PD deficient (Table 1). Six of 14 heterozygous G6PD Aures subjects presented G6PD intermediate (mean 3.12 U/gHb; 95% CI: 2.66–3.58 U/gHb). whereas the remaining 8 females showed normal G6PD enzyme activity (mean 6.48 U/gHb; 95% CI: 5.44–7.51 U/gHb).

## Discussion

*G6PD* mutations are distributed worldwide and particularly widespread in malaria endemic regions (15, 29). Knowledge of the prevalence of G6PD variants among different ethnic groups is limited. The present study is the first to report the prevalence of G6PD deficiency and *G6PD* mutations in the Mon-Khmer or Lao Theung ethnic group. The prevalence of G6PD deficiency reported in this study (9.13%) is higher than that previously reported for unspecified ethnic Lao populations (3.3%, 6.2% and 7.2%) [20, 23, 25] (Table 2).

**Table 2:** Prevalence and molecular characteristics of G6PD deficiency in Lao PDR

	This study	Hsia et al. 1993 (22)	Iwai et al. 2001 (20)	Kanchanavithayakul et al. 2017 (21)	Lover et al. 2018 (25)	Bancone et al. 2019 (23)	Ong et al. 2019 (24)
G6PD enzyme deficient							
Male %	5/78 (6.41%)	15/74 (20.3%)	21/291 (7.2%)	31/141 (21.98%)	30/910 3.30%	106/1211 (8.8%)	ND
Female %	18/174 (10.34%)	ND	ND	10/89 (11.24%)	ND	79/1764 (4.50%)	ND
Total %	23/252 (9.13%)	15/74 (20.27%)	21/291 (7.2%)	41/230 (17.8%)	30/910 3.30%	185/2978 (6.21%)	ND
*G6PD* mutation							
Gaohe 95A > G	ND	–	–	ND	ND	ND	ND
Aures 143 T > C	17 (6.75%)	ND	ND	ND	ND	ND	ND
Asahi 202 G > A	ND	–	ND	ND	ND	ND	ND
A 376 A > G	ND	–	ND	ND	ND	ND	ND
Vanua Lava 383 T > C	–	ND	–	ND	ND	ND	ND
Mahidol 487G > A	2 (0.79%)	2 (2.7%)	–	–	1 (0.11%)	4 (0.1%)	ND
Chinese-3 493 A > G	ND	-	ND	ND	ND	ND	ND
Mediterranean 563C > T	–	ND	ND	ND	–	ND	ND
Coimbra 592C > T	–	ND	–	–	–	ND	ND
Chinese-1 835 A > T	ND	–	ND	ND	ND	ND	ND
Viangchan 871G > A with nt1311T	1 (0.40) %)	5 (6.8%)	9 (3.1%)	15 (6.5%)	11 (1.21%)	115 (3.9%)	90 (4.4%)
Jammu 871G > A with nt1311C	2 (0.79%)	ND	ND	–	ND	ND	ND
Chatham 1003 G > A	ND	–	–	ND	ND	ND	ND
Chinese-5 1024 C > T	ND	ND	ND	–	ND	ND	ND
Surabaya 1291G > A	ND	ND	–	ND	ND	ND	ND
Union 1360C > T	3 (1.19%)	1 (1.4%)	–	1 (0.4%)	14 (1.54%)	9 (0.3%)	ND
Canton 1376G > T	1 (0.4%)	–	–	4 (1.7%)	4 (0.44%)	1 (0.03%)	ND
Kaiping 1388G > A	2 (0.79%)	1 (1.4%)	–	2 (0.9%)	ND	4 (0.1%)	ND
Quing Yan 392G > T	1 (0.4%)	ND	ND	–	ND	6 (0.2%)	ND

*ND* not done

However, the prevalence of G6PD deficiency in Lao populations was lower than that reported in neighboring Southeast Asian populations, including Mon (12%) [17, 30], Karen and Burman (13.7%) [21], Thai (11.1%) [18], and Cambodian (26.1%) [19] populations. The prevalence of G6PD deficiency in Laotians had previously been reported in an unspecified ethnic population, and it was suspected that the majority of the studied subjects were Lao Loum, which are the main Laotian ethnic group. In the beginning of *G6PD* mutation screening, the study of *G6PD* mutations focused only on the detection of the G6PD Viangchan mutation or identified the *G6PD* mutation only in males that presented with severe haemolytic anaemia [20, 21, 23]. Only six previous studies (Table 2) have revealed that the prevalence of G6PD deficiency in the Lao population ranges from 3.30 to 21.98% in males [20–23, 25] and 4.50-11.24% in females [21, 23]. This study revealed severe G6PD deficiency more frequently in males than in females, whereas intermediate G6PD deficiencies were more common in females than in males. Although the females with heterozygous *G6PD* mutation have sufficient enzyme activity, they can pass an X-linked G6PD mutation to all of their sons and daughters along with a risk of developing the symptoms associated with a severe G6PD deficiency.

Regarding the mutation characteristic of the *G6PD* gene, eight *G6PD* mutations were detected in this Lao Theung group. A comparison of the *G6PD* mutation data collected in this and previous studies of non-specified Lao ethnic populations is summarized in Table 2. The most common *G6PD* mutation in the Lao Theung population was G6PD Aures c.143 T > C (6.75%); this result is different from that in a previous report, which found that the G6PD Viangchan mutation was the most common mutation in the Laotian population (1.21–6.76%) [20–25]. The G6PD Jammu mutation is an 871G > A mutation identical to the G6PD Viangchan mutation but different from the polymorphism at nucleotide 1311 (nt1311C) detected in this study, whereas the previous report found only G6PD Viangchan in the Laotian population [21]. The polymorphisms nt1311C and IVS11 nt93T were randomly detected in this group, different from those reported in other Lao populations [21], the Thai population (nt1311T) [18] and the Chinese population (nt1311T) [31]. These data imply that the Mon-Khmer or Lao Theung and Lao Loum groups have different origins. For neighboring Southeast Asian countries, G6PD Mahidol is commonly found in Burmese (18.21%) [23], while G6PD Viangchan is commonly found in Thais (6.0%) [18], Cambodians (17.72%) [19] and Vietnamese (26.7%) [32], but not in the Lao Theung Mon-Khmer population. In addition, we rarely detected the G6PD Canton, G6PD Kaiping and G6PD Union mutations, which are prevalent in South China [33]. The G6PD Mediterranean, G6PD Vanua Lava and G6PD Coimbra mutations were not found in this population. The *G6PD* mutation was not identified in five G6PD-deficient participants. The mutation site of those 5 cases may exist in the intron region, which could not be analyzed by our method.

The G6PD Aures mutation is commonly detected in Mediterranean populations, such as the Saudi Arabian (11.2–20%) [34–36], United Arab Emirates (11.9%) [37] and Kuwaiti (3.73%)[38]. The G6PD Aures was detected in 0.7–4.3%of Thai populations [39, 40]. This mutation results in the substitution of amino acid 48 from isoleucine to threonine. The WHO has classified the G6PD Aures mutation as a class III mutation [41]. A previous report described the person who carried this mutation as having mild G6PD deficiency [42].

However, severe G6PD deficiency was observed for the first time for the Aures mutation in this study. The hemizygous males and homozygous females for the G6PD Aures mutation presented with a severe G6PD deficiency, whereas the phenotype of G6PD Aures heterozygous females varied from normal to moderately G6PD deficient. The G6PD Aures is located between the sites of G6PD Vietnam-1 and Vietnam-2/Bahia, near G6PD Rignano, which is distant from the protein domain for NADP-1 binding [43]. The mutation was predicted to affect the mini-instability of protein domain structure for binding with NADP-1 and to subsequently cause mild G6PD deficiency. A previous report described the person who carried this mutation as having mild G6PD deficiency [42]. The hemizygous males or homozygous females for the G6PD Aures mutation presented with a significant severe G6PD deficiency, whereas the heterozygous females for G6PD Aures had G6PD intermediate and normal G6PD enzyme activity.

The G6PD Aures mutations were detected in both male and female participants. As the inheritance of G6PD is X-linked, it is very important to address the finding that both homozygous and heterozygous G6PD females can pass the abnormal gene to their male child, leading to the risk of clinically presenting the G6PD Aures mutation. The knowledge about the effect of the G6PD Aures mutation is now still limited, and it is very important to test the haemolytic risk of anti-malarial drugs and other oxidative damage related to the G6PD Aures mutation in further studies as the Lao Theung people stay around the forest areas and have a risk of malaria infections.

This study reports the development of ARMS-PCR, which can detect the G6PD Aures in one PCR-electrophoresis step. The diagnosis of G6PD Aures could be alternatively performed by PCR sequencing analysis, as the *G6PD* gene flanking the G6PD Aures mutation site must be amplified by PCR, purified and sent for sequencing analysis. Compared to the PCR sequencing method, the ARMS-PCR developed in this study can show a positive or negative G6PD Aures result in a shorter time with lower costs; however, this method cannot be used to determine the zygocity of the G6PD Aures. By using the ARMS-PCR method developed in this study, the G6PD Aures mutation was additionally detected in 7 female participants. This new method should be applicable in routine clinical practice for the detection of the G6PD Aures.

## Conclusions

The *G6PD* mutation pattern identified in the Mon-Khmer group is quite homogenous and unique, predominantly involving the G6PD Aures mutation. This finding shows a founder effect in this population. The hemizygous males or homozygous females for the G6PD Aures mutation presented with a significant severe G6PD deficiency. Malaria infection, dengue fever and typhoid fever are prevalent in Lao PDR and knowing the G6PD status in local health areas, especially Lao Theung group, helps improve the management of acute haemolysis related to these infections.

## Data Availability

All data generated or analysed during this study are included in this published article.

## Abbreviations

G6PD: Glucose 6˗phosphate dehydrogenase
PCR: Polymerase chain reaction
ARMS: Amplification refractory mutation system
